# Prophylactic Swallowing Exercises in Patients with Laryngeal Cancer Who Underwent Total Laryngectomy—A Randomized Trial

**DOI:** 10.3390/curroncol31110506

**Published:** 2024-11-02

**Authors:** Elena Teodora Schipor-Diaconu, Raluca Grigore, Paula Luiza Bejenaru, Catrinel Beatrice Simion-Antonie, Bianca Petra Taher, Simona Andreea Rujan, Anca Ionela Cirstea, Raluca Andreea Iftimie, Ruxandra Ioana Stancalie-Nedelcu

**Affiliations:** 1 Department of Otorhinolaryngology, Ophtalmology, Faculty of Medicine, “Carol Davila” University of Medicine and Pharmacy, 050474 Bucharest, Romania; elena-teodora.diaconu@drd.umfcd.ro (E.T.S.-D.); anca-ionela.cirstea@drd.umfcd.ro (A.I.C.); 2 Department of ENT, Head and Neck Surgery, Coltea Clinical Hospital, 030167 Bucharest, Romania; 3 Department of ENT, Head and Neck Surgery, Emergency Universitary Hospital Bucharest, 050098 Bucharest, Romania; 4 Ophtalmological Emergencies Clinical Hospital, 010464 Bucharest, Romania

**Keywords:** total laryngectomy, swallowing exercises, swallowing function, quality of life, rehabilitation

## Abstract

Objectives: This study aims to determine the efficacy of prophylactic swallowing exercises on swallowing function in patients undergoing total laryngectomy for laryngeal cancer. Methods: The design was a randomized controlled trial set in one tertiary care academic medical center. A total of 92 patients undergoing total laryngectomy for stages III and IV laryngeal cancer performed five targeted swallowing exercises for a period of three months after their surgery, starting two weeks after the surgery. Weekly swallowing therapy sessions were held with the patients in order to encourage adherence and proper technique. The controls received no preventive exercise and were referred for swallowing treatment following the surgery, as well as radiation therapy if necessary. The Functional Oral Intake Scale (FOIS) and the Performance Status Scale for Head and Neck Cancer Patients (PSS-H&N) were used to measure swallowing function at the baseline, one week following the surgery, and three, six, nine, and twelve months following the surgery. Results: Right after the surgery, there were no statistically significant variations between the intervention and control groups in the FOIS scores (*p* value = 0.64), the Eating in Public subscale scores (*p* value = 1) and Normalcy of Diet subscale scores (*p *= 0.33) of the PSS-H&N. The scores were significantly better among the intervention patients at months 3, 6, 9, and 12 for all the scores, with *p* values smaller than 0.000. Conclusions: Although not immediately following the surgery, the patients who engaged in prophylactic swallowing exercises showed improvements in their ability to swallow at 3, 6, 9, and 12 months following their procedure.

## 1. Introduction

**Figure 1:**
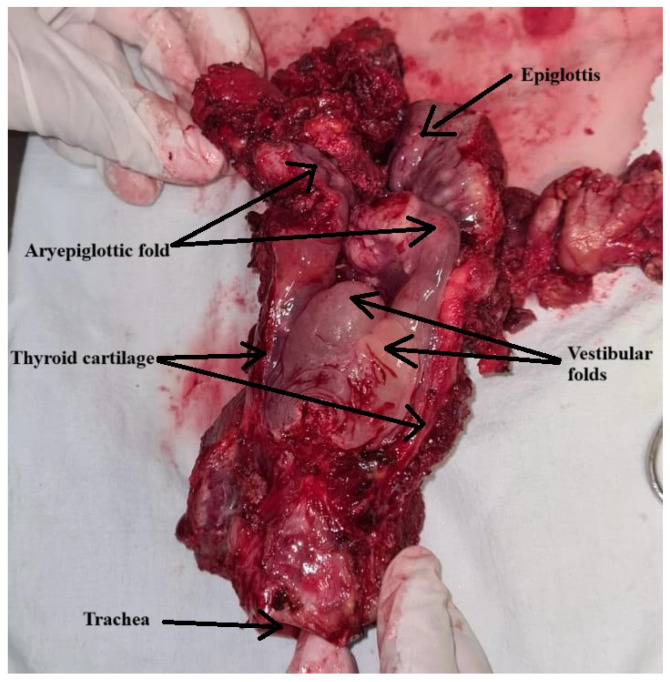
Total laryngectomy piece with a stage IV tumor.

The incidence and mortality rates of laryngeal cancer have been slightly decreasing for the past 10 years in European countries [1]. For Romania, laryngeal cancer is the 16th most common type of cancer, with an incidence of 1804 patients, accounting for 1.7% of all cancers [2]. The oncologic control of laryngeal carcinoma is the main objective of its treatment. In order to maintain a good quality of life, preserving speech and swallowing abilities are secondary but very important objectives. Laryngeal carcinomas can be treated with radiation therapy, chemotherapy, surgery, or a mix of these. Treatment for laryngeal cancer should be determined by the knowledge of an interprofessional team, such as that found in a multidisciplinary clinic or on a tumor board. The type, location, and function of the larynx, as well as the patient’s medical and social comorbidities, all influence the course of the treatment. Also, when nonsurgical treatments of laryngeal cancer have failed or recurred, total laryngectomy is the standard of care for surgical salvage; it is also frequently used as the main treatment for advanced-stage laryngeal malignancy [3]. Advanced-stage (III and IV) tumors require multimodality treatment, which may consist of surgery followed by radiation therapy [4]. In Figure 1, we have a total laryngectomy piece with a stage IV squamous cell carcinoma (T4a, N2c, M0). It can be seen that the whole larynx is resected; the tumor involves the epiglottis and right aryepiglottic fold, protrudes through the thyroid cartilage on the right side, and also involves the first tracheal ring. Considering the extent of the tumor, only a total laryngectomy could be performed. The structures that are most important for the swallowing function are the epiglottis, the aryepiglottic folds, and also the vestibular folds, but in this case, they could not be spared. Also, the superior laryngeal nerve is important as it innervates the cricothyroid muscle, thus providing a good swallowing function; in this case, it could not be spared. For a good swallowing function after surgery, it is important that the pharyngeal wall is well preserved so that the suture is made without tension. For all the patients in this study, the pharyngeal wall suture was tension-free.

Total laryngectomy can lead to numerous changes, the most obvious being the loss of the natural voice, but also the loss of upper airway functions (the moistening, heating, and filtering of air), resulting in pulmonary problems and the loss of olfaction. After the surgery, the patient has to adapt to the altered anatomy and its lifelong consequences, leading to physical, emotional, psychological, and social changes that affect their average daily functioning and quality of life [5].

The altered physiology and biomechanics of swallowing are another significant effect. While after such major surgery people expect and become accustomed to some degree of diminished swallowing functioning, studies show that long-term self-reported swallowing problems can appear in as much as 72% of patients after TL (total laryngectomy) [5]. The estimates of the frequency of swallowing problems (dysphagia) after TL usually range from 17 to 70%. The characteristics most frequently considered distressful by patients were having to take longer to be able to swallow, needing liquids to wash down a bolus, and avoiding particular food consistencies [6]. Dysphagia can also lead to malnutrition in patients with TL. Malnutrition has long been identified as an important prognostic factor, associated with a poorer quality of life and reduced survival in patients, as well as being associated with post-operative complications including the development of pharyngo-cutaneous fistula, infection, and delayed wound healing [7]. Being so frequent amongst patients with TL and leading to numerous complications, dysphagia becomes one of the most important problems which should be addressed in order to maintain a good quality of life. Swallowing exercises targeted at particular swallowing deficits can be used to improve the mobility and motility of vital swallowing structures. There were five intervention swallowing exercises chosen because each has been shown to improve swallowing function.

## 2. Materials and Methods

From 1 April 2023 to 1 April 2024, we recruited 96 consecutive patients with stages III and IV laryngeal cancer. The objective was to compare the mean outcomes of a treatment group and a control group with the effect size being the expected difference in the means between the treatment and control groups. The significance level (α), as is common, was set at 0.05, and the probability of correctly rejecting the null hypothesis when it is false was set at 0.80. With a total sample size of 96 (48 per group), the effect size that can be detected with 80% power at a 5% significance level, given a standard deviation of 10 units, is approximately 5.71 units. As the effect size that we were aiming to detect was larger than 5.71, a sample size of 96 patients (48 per group) was considered sufficient.

**Chart 1:**
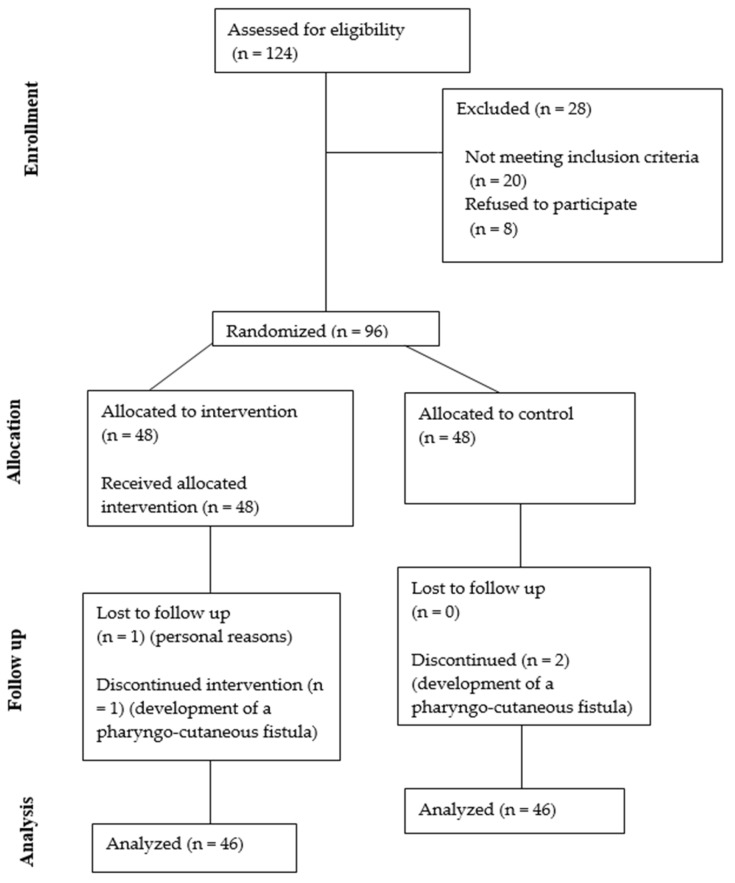
Process of enrollment, allocation, follow up and analysis of the sample.

We included patients that were diagnosed at our center, but also patients that came with a diagnosis from other centers. All the patients went through a case analysis performed by our tumor board. The tumor board analyzed the stage of the tumor, the presence or absence of distant metastases, and the histopathology of the tumor, and only patients with laryngeal squamous cell carcinoma stages 3 and 4 who were suitable for surgery (total laryngectomy with selective neck dissection) were included in this study. Patients that could not be operated on were referred to radiotherapy and/or chemotherapy. Also, patients who required extensive surgery such as total pharyngo-laryngectomy and/or esophagectomy were excluded from this study. We also excluded patients who had previously undergone radiation treatment as this produces changes which can interfere with the quality of swallowing amongst patients. Patients with a history of neurological conditions that might impair swallowing ability were also excluded. All the patients also had to be mentally and cognitively capable of following instructions with several steps and comprehending how to respond to certain questions on a questionnaire. One patient dropped out of this study, and three developed a pharyngo-cutaneous fistula, so they were excluded from this study. The remaining sample size was 92 patients. The Coltea Clinical Hospital’s institutional ethics committee board approved this study on 20 March 2023, and issued the approval number 5820/abcc. Every patient gave their informed permission. The intervention or control treatments were randomly assigned to the patients (Chart 1).

The objective of this study was to assess whether prophylactic swallowing exercises initiated immediately after surgery can lead to an improvement in the swallowing function in patients undergoing total laryngectomy for laryngeal cancer.

### 2.1. Intervention Group

Prophylactic swallowing exercises were the intervention; the patients were encouraged to begin these exercises two weeks following the surgery and to continue them for three months.

#### 2.1.1. Effortful Swallow (ES)

**Figure 2:**
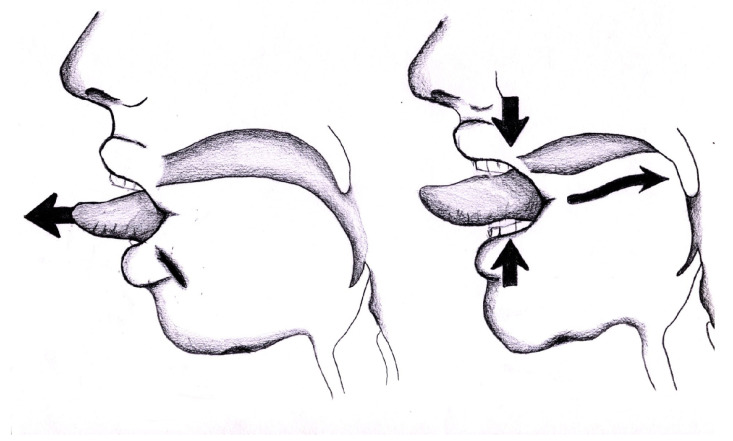
The effortful swallow maneuver.

The goal of the effortful swallow (ES) technique is to push and swallow with enough force to aid in bolus clearance while applying increased pressure to the bolus, and it is known for its instantaneous effect [8]. An easy way to perform it is to stick the tongue out and hold it between teeth while swallowing (Figure 2). The purpose of the effortful swallow maneuver is to increase pressure on the bolus by enhancing the contact between the posterior pharyngeal wall and the base of the tongue during swallowing. Initially, the effortful swallow was suggested as a compensatory technique to improve bolus clearance in the vallecula by facilitating bolus flow into the pharynx. However, because of its ability to modify the physiological elements of swallowing, the ES is also employed as a therapeutic or rehabilitative treatment. The effortful swallow has multiple physiological effects, and because it is a simple maneuver, it is frequently used in clinical practice. All the sensor locations with both the saliva and water trials and across different ages showed that the creation of the tongue-to-palate maximum pressure was larger during the effortful swallow than during normal swallowing. This finding is similar across studies [9].

#### 2.1.2. Tongue Pull Back (TPB) Exercise 

**Figure 3:**
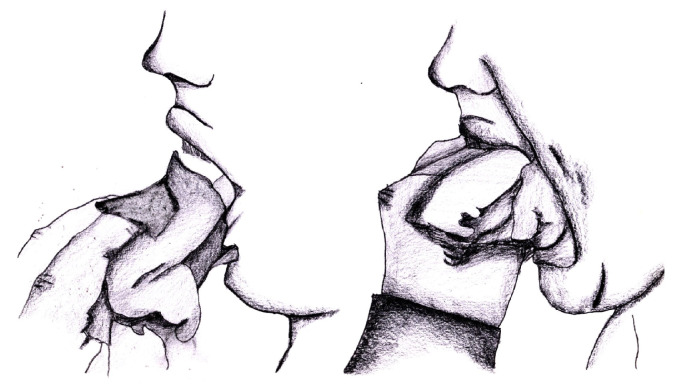
The tongue pull back exercise.

A number of intricate sensory–motor processes work together during the pharyngeal swallow to convey a bolus smoothly and successfully through the pharynx and into the esophagus. Pharyngeal constrictors contract sequentially, the tongue base retracts, and the upper esophageal sphincter (UES) opens. In the swallowing process, the purpose of tongue base retraction is to shift the base of the tongue posteriorly to meet the posterior pharyngeal wall, which travels both superiorly and anteriorly. In order to force the bolus through the pharynx and into the esophagus during swallowing, the posterior movement of the tongue base and superior–anterior movement of the posterior pharyngeal wall work together to raise pharyngeal pressure [10]. A deficit in tongue base retraction causes residue in the pharynx and is prevalent in individuals with dysphagia [11]. Regarding the precise muscles involved in tongue base retraction, the research is ambiguous. When discussing anatomy, the majority of sources mention the intrinsic and extrinsic lingual muscles. However, when describing the tongue base’s propulsive activity during the pharyngeal swallow, they typically refer to the “tongue base” or “posterior tongue” [10,12]. The most often recognized muscles involved in tongue base retraction are the hyoglossus [13], genioglossus [14,15], and styloglossus [13,15]. Some publications also list the mylohyoid, digastric, and geniohyoid muscles [16]. The classic exercise for strengthening the tongue base is the tongue retraction, in which the patient pulls back the tongue as far is it can go, like trying to touch the back of the tongue to the roof of the mouth. Studies have shown that adding resistance to the TPB exercise can increase its efficiency [17]. One method described in the literature is the finger-resisted TPB exercise. Using a piece of sterile gauze to hold the tongue between the fingers, the participant has to pull the tongue into the mouth. The thumb is positioned beneath the tongue, while the index and middle fingers are positioned above. During the exercise, participants are advised to open their lips wide, allowing their fingers to enter, while they pull back their tongue. They are told to offer some resistance, but not so much that it would limit range of motion or cause them to lose their tongue hold. They are told not to force the tongue back (Figure 3).

#### 2.1.3. Chin Tuck Against Resistance (CTAR) Exercise 

**Figure 4:**
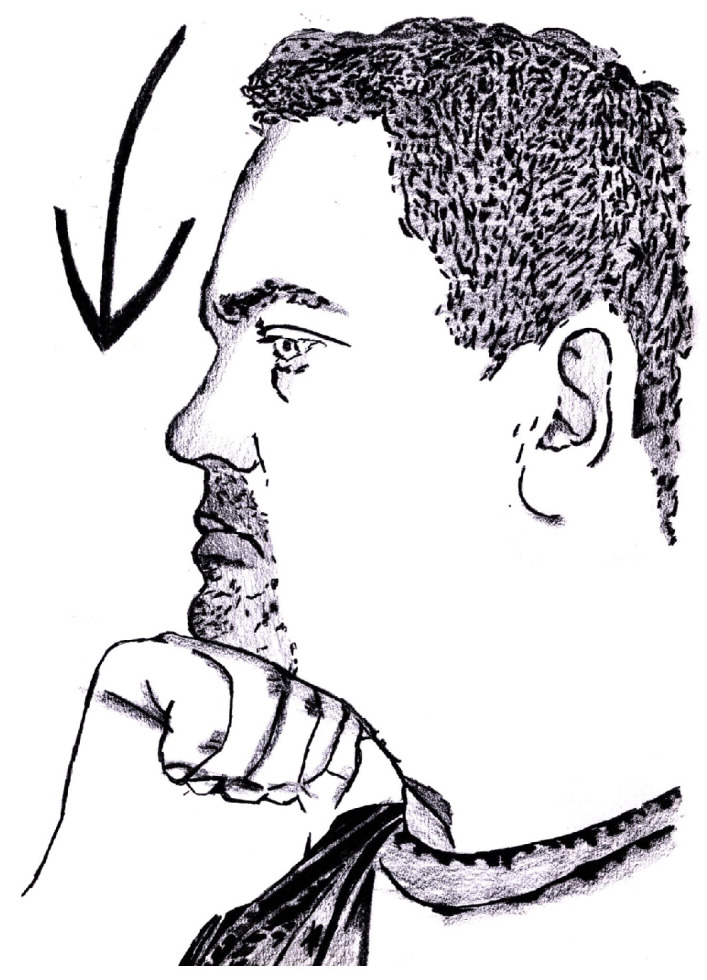
Chin tuck against resistance exercise.

By strengthening the swallowing muscles, the chin tuck against resistance technique helps in swallowing. It especially targets the suprahyoid muscles. The CTAR exercise involves the patient pulling their chin down toward their upper chest against a resistance like a rubber ball or other such object or even the patient’s hand (Figure 4). This exercise is performed while sitting. It is easy to modify CTAR exercises to target different muscles and enhance muscle coordination during swallowing by varying the resistance level or position. It is critical to choose the right resistance level for the patient’s physical state and treatment objectives. The phases of the CTAR exercise are as follows:

1. Preparation—the patient sits comfortably.
2. Positioning—in our case, the patient places their hand under their chin.
3. Chin tuck—the patient tucks the chin down toward the chest.
4. Resistance—the patient applies resistance with their hand. Typically, the resistance is kept for 5–10 s.
5. Rest and repeat—after a brief period of relaxation, the activity is repeated. It is often advised to carry out several sets of repetitions each day [18].

Tongue pressure is enhanced by the CTAR exercise, which is beneficial for a healthy swallowing function. This exercise also helps to build muscle endurance and strength, which enhances the overall swallow function. Comparing chin tuck against resistance exercises to traditional therapeutic approaches alone reveals a considerable improvement in the tongue pressure and overall swallow performance. The correct patterns of muscular activation during swallowing are reinforced through the repetition of CTAR exercises [19].

#### 2.1.4. The Head Lift (HL) Exercise 

**Figure 5:**
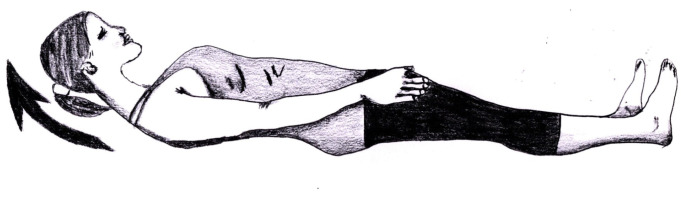
The head lift exercise.

Using the knowledge that the pull of the thyrohyoid, mylohyoid, geniohyoid, and anterior belly of the digastric muscles contracting causes the following opening of the upper esophageal sphincter, the head lift exercise, also referred to as the Shaker exercise, is defined. Enhancing the muscles’ strength and endurance is the goal, as it will increase the upper esophageal sphincter’s opening width. The workout comprises an isometric high-intensity head-raising that includes three head raises held for 60 s each, with a 60 s rest period between them and an isokinetic low-intensity segment consisting of 30 successive head lifts at a steady pace without holding (Figure 5). The goal of it is to raise the anteroposterior diameter and the cross-sectional area of the opening of the upper esophageal sphincter. This is a non-invasive exercise designed specifically for people with dysphagia. It is a substitute for invasive procedures like botulinum toxin injections or cricopharyngeal myotomies [20]. The HL exercise is an extremely difficult exercise for physically fragile persons, such as the elderly and stroke patients, even if it is beneficial for improving the swallowing function in dysphagia patients. A person who finds it difficult to physically change positions cannot readily complete this exercise, mostly because it requires being in the supine position. Additionally, it can wear down the neck’s muscles, particularly the sternocleidomastoid. Frequent exposure to muscular exhaustion can result in transient pain and discomfort. This lowers compliance, being one of the reasons why patients discontinue their treatment, so performing this exercise assisted should happen as often as possible [21].

#### 2.1.5. Resistive Jaw-Opening (RJO) Exercise 

**Figure 6:**
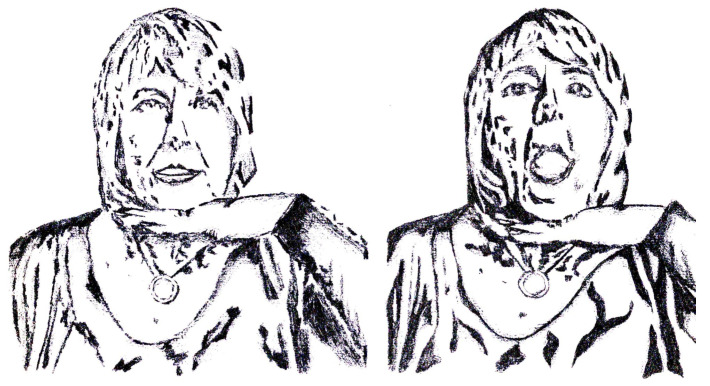
Jaw opening against resistance.

This is an exercise that strengthens the temporomandibular joint muscles and also activates the suprahyoid muscle. To produce resistance, the thumb is placed directly beneath the chin, and then the patient presses upward while opening the mouth. While keeping the upward pressure, the patient progressively seals their mouth while resisting the upward pressing (Figure 6). The practice entails fully expanding the jaw and holding it there for ten seconds. The exercise has to be performed every day, with two sets of exercises of five repetitions each with a ten second pause between every repetition. Furthermore, Wada et al. showed that the RJO exercise enhanced the upper esophageal sphincter opening in dysphagia patients after four weeks of use [22]. The isometric maximal tongue base pressure and tongue endurance have both been demonstrated to considerably increase after this workout [23]. Additionally, it can thicken the mylohyoid and digastric muscles, which is significant because muscle thickness and contractile force are closely correlated. In order to induce changes in muscle physiology, such as changes in muscle thickness through resistance training, an adequate resistance and prolonged exercise duration are essential. To specifically induce skeletal muscle physiologic changes, such as muscular hypertrophy, the training should be performed for a minimum of 6 to 8 weeks. The RJO exercise has the benefit of being less strenuous than the HL exercise, but it also carries the risk of temporomandibular joint pain and dislocation issues. Consequently, it is not advised for individuals with severe temporomandibular joint issues, and it also necessitates paying close attention to how the temporomandibular joint feels and wears out during the exercise [21].

### 2.2. Control Group

The controls received no preventive exercises and were referred for swallowing treatment following the surgery on an “as needed” basis as swallowing issues appeared in patients.

**Figure 7:**
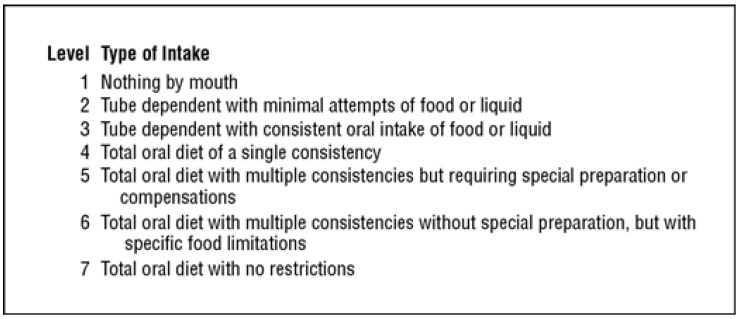
Functional Oral Intake Scale.

**Figure 8:**
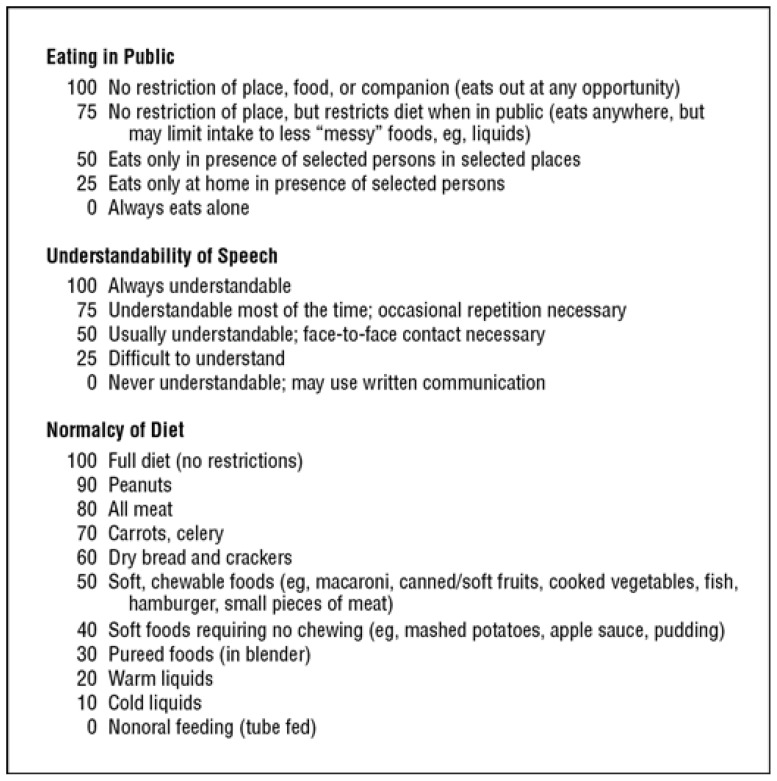
Performance Status Scale for Head and Neck Cancer patients.

Weekly swallowing therapy sessions were attended by the patients in order to encourage adherence and proper technique. The standard of care was used as the control treatment, which entailed referring patients who had dysphagic symptoms after the cancer treatment was finished to a head and neck speech pathologist for evaluation and treatment related to swallowing. Five intervention swallowing exercises were selected because research has demonstrated that they can all help people with dysphagia improve their ability to swallow. They included the resistive jaw-opening exercise, the head lift exercise, the chin tuck against resistance, the tongue pull back exercise, and the effortful swallow. To further promote adherence to the swallowing exercise routine and to document the names of those who were unable to complete it, the patients were advised to keep a daily performance diary. Written instructions on how to carry out the individual swallowing exercises were given to each patient. Two distinct swallowing-specific scales that also addressed some swallowing-related quality of life issues were used to perform functional swallowing assessments. These comprised the Functional Oral Intake Scale (FOIS) [24] (Figure 7) and the Performance Status Scale for Head and Neck Cancer patients (PSS-H&N) [25] (Figure 8). All the research participants were evaluated by a clinician who was specially trained in the use of these scales and who was blinded to the intervention assignment one week following the surgery to establish a baseline and then at three, six, nine, and twelve months following the surgery. Three distinct subscales make up the short, clinician-rated PSS-H&N: Normalcy of Diet, Understandability of Speech, and Eating in Public. Every subscale has a value between 0 and 100; a higher score denotes a better function. The Eating in Public subscale records the patient’s capacity to share a meal with others in public, which helps to address swallowing-related quality of life problems. The interviewer’s ability to comprehend the patient’s speech is rated on the Understandability of Speech subscale. The patient’s ability to handle a regular diet is measured by the Normalcy of Diet subscale. Ten food categories are ranked on this subscale, with easier-to-eat alternatives at the bottom and more difficult-to-eat options at the top. This scale is sensitive to functional variations in a wide range of head and neck cancer patients and has been demonstrated to be reliable among raters [24]. The Understandability of Speech subscale of the PSS-H&N was not examined since the ability of speech is lost for these patients for variable amounts of time depending on the method chosen for speech rehabilitation. A 7-point oral dietary tolerance scale is called the FOIS. It varies from total reliance on PEG or a nasogastric tube (1) to the tolerance of an unrestricted oral diet (7). It offers crucial details regarding the kinds of adjustments or restrictions that patients must make to their oral diet and whether they require tube-based nutritional supplements [25].

### 2.3. Statistical Analysis

We used Excel for analyzing the data and used the *t*-test: Paired Two Sample for Means. We looked at variations in the therapy assignment based on the patient characteristics. The PSS-H&N and FOIS scores were treated as continuous variables. Intention-to-treat analyses were performed to look at the outcomes for both the intervention and control patients. We compared the scores and reported the differences in the scores at each time point following the baseline (at 3, 6, 9, and 12 months after the surgery). The threshold for significance was chosen at *p* < 0.05 (two-tailed).

### 2.4. Descriptive Analysis

In Table 1 is described the analysis of the tumor stage, age and BMI of the sample.

## 3. Results

Out of all the patients, ninety-six consented to take part, one patient dropped out, and three patients developed a pharyngo-cutaneous fistula, so the sample size was 92 patients. The participants in this study had a mean age of 63.98 years, 80% of whom were male. Regarding age and sex, there were no appreciable differences between the intervention and control groups; the *p* value for age was 0.94 and for sex was 0.64. The BMI was also calculated for all the patients and there were no significant differences between the control and the intervention group; the *p* value was 0.25. For the majority of the patients in the intervention and control groups, the baseline scores on all the evaluations were identical (Table 1).

### Swallowing Function and Swallowing-Related Quality of Life (QOL)

The swallowing-related QOL and Normalcy of Diet scores as measured by the Eating in Public and Normalcy of Diet subscales of the PSS-H&N were quite low at the baseline for the patients in both the intervention and control groups, with a range from 0 to 25 in both groups. There were no statistically significant differences in the Eating in Public subscale scores between the patients immediately after the surgery (*p* value = 1); however, these scores improved after 3, 6, 9, and 12 months for the intervention group. The scores were significantly better among the intervention patients at months 3 (*p* value < 0.000), 6 (*p* value < 0.000), 9 (*p* value < 0.000), and 12 (*p* value < 0.000).

Likewise, there were no statistically significant differences in the Normalcy of Diet subscale of the PSS-H&N scores between the patients from the intervention group and the control immediately after the surgery (*p* = 0.33). The range was from 0 to 20 for both the intervention and control groups at the baseline. The scores improved for the intervention patients relative to the controls at months 3 (*p* value < 0.000), 6 (*p* value < 0.000), 9 (*p* value < 0.000), and 12 (*p* value < 0.000).

The oral dietary tolerance (FOIS score) followed a similar pattern among the intervention and control patients. There were no statistically significant differences in the FOIS scores between the individuals in the intervention group and the control immediately after the surgery (range from 1 to 3 in both groups, *p* value = 0.64). However, the patients in the intervention group have significantly improved FOIS scores at months 3 (*p* value < 0.000), 6 (*p* value < 0.000), 9 (*p* value < 0.000), and 12 (*p* value < 0.000) (Table 2).

## 4. Discussion

The patients randomized to perform prophylactic swallowing exercises had functional swallowing and swallowing-related QOL outcomes that were significantly better than those of the patients who were referred for swallowing assessment and treatment on an as-needed basis after completing their treatment, according to this study of patients with laryngeal cancer undergoing total laryngectomy. This study’s limited sample size could make it harder to identify differences and result in just a partial reflection of the real variations. To address this lack of difference more conclusively, greater research on the effects of preventive swallowing exercises over time may be beneficial. It is yet unclear if the control patients could catch up to the intervention patients in time and if the control patients with persistent dysphagic symptoms who received swallowing evaluation and treatment after the treatment for cancer was concluded were able to improve their swallowing function to the level observed in the patients who had completed the prophylactic swallowing exercise intervention. More research involving a larger patient population over a longer period of time is required.

Through a prospective randomized controlled experiment, we examined the impact of preventive swallowing exercises on swallowing outcomes in patients undergoing total laryngectomy.

Exercise improves swallowing function; however, the exact process is unclear. Patients may exhibit edematous tissue and a progressive development of fibrosis. For some people, fibrosis may manifest years after the end of their cancer treatment. In either case, fibrosis causes problems with the swallowing structures’ ability to move and coordinate, which in turn disrupts the effective and efficient bolus transport required for swallowing function [26]. Exercise may help to reduce some of the parameters associated with fibrosis, according to a recent study on the impact of exercise on wound healing and inflammation reduction in mice [27]. Furthermore, by strengthening the nonfibrotic tissue to make up for the fibrotic structures’ lack of mobility, the training of the swallowing structures may also aid [28].

Also, these types of exercises improve the muscle tone and strength and altogether the functionality of the tongue. The anatomy and physiology of the swallowing mechanism undergo significant changes after a total laryngectomy, and the tongue, which plays a crucial role in the oral phase of swallowing, needs to adapt. The capacity to adapt is primarily linked to the tongue’s trophism. The tongue takes on a primary role in controlling boluses, propelling them forward, and starting the pharyngeal phase of swallowing. The tongue’s ability to collect and move food from the oral cavity into the oropharynx depends on a healthy trophism. Exercises enhance tongue strength, coordination, and endurance, being associated with higher tongue forces at all ages, directly impacting the quality of swallowing [29].

A 2007 abstract from the Dysphagia Research Society Meeting by Carnaby-Mann et al. that examined the impact of a behavioral swallowing training program on the preservation of swallowing-related muscle composition is also worth mentioning. When comparing patients who received behavioral swallowing treatment during head and neck cancer treatment to controls, they discovered that the former group had a higher degree of swallowing muscle preservation [30].

Established swallowing exercises were included for the intervention group, and the combination of exercises was chosen to improve bolus transport, which is widely acknowledged as the main dysphagic consequence that affects patients. We also used two validated measures of swallowing function that were administered by clinicians. These measures addressed the swallowing-related quality of life and the issues of being able to eat outside the home and with others, as well as providing a detailed description of the patients’ oral tolerance and intake and the need for complete or partial PEG use [31].

However, it is important to note some of this study’s limitations. We did not employ an analysis of some of the patients’ parameters, such as the anthropometric parameters or the type of diet or nutritional status of the patients before the surgery, subgroup analyses, and a multivariate analysis. Only the BMI is insufficient to characterize the whole status of the patients, and this could have added to a better understanding of how the exercises affect certain types of patients, which types benefit more from performing the exercises, and how we can adapt them to patients that have a lower nutritional status, so a higher risk of developing malnutrition.

We did not employ video–fluoroscopic assessments, which could have yielded a more accurate gauge of the exercises’ impact on the swallowing function. Most practicing swallowing clinicians consider video–fluoroscopy, also called a modified barium swallowing examination, to be the preferred tool because it allows the real-time visualization of bolus flow in relation to structural movement throughout the upper aerodigestive tract. Additionally, physicians can watch how different bolus textures, volumes, and compensatory techniques affect the physiology of swallowing [32]. Even though the examination is clinically useful, doctors need to be aware that a patient’s performance during the examination might not be totally indicative of how they typically eat and drink. Treatment can be applied systematically during and after the evaluation in accordance with the physiologic swallowing problem when the video–fluoroscopy procedure is standardized, interpreted, and reported by skilled clinicians utilizing standardized and validated metrics [33]. Video–fluoroscopic swallowing examinations at the same time points could be beneficial for future research to monitor any changes in swallowing function over time during the course of swallowing treatment and to assess the progression of the condition [33].

A larger sample size would also have been necessary to address the crucial question of how much and how often the exercises must be performed in order to produce a benefit for swallowing. It may have also allowed us to predict which patients would have benefited more from prophylactic swallowing exercises. Furthermore, as mentioned, the small sample size might have made it more difficult for us to determine the precise amount of the variations that were seen and to find statistically significant differences in the swallowing function.

Even though this study’s outcomes are positive, we still need to be aware of the substantial toll that undergoing total laryngectomy for the treatment of laryngeal cancer has on our patients. Although the results of swallowing following treatment are obviously improved by instituting a strict preventive swallowing regimen, we must remember the additional burden this places on each patient and continue to be mindful of how much some patients can or cannot handle.

## 5. Conclusions

In summary, at three, six, nine, and twelve months following the cancer treatment, the patients who engaged in prophylactic swallowing exercises demonstrated significantly improved swallowing outcomes. To build on these results and offer a more robust analysis of the impact of preventive swallowing exercises on these patients, future research with a bigger sample size is required.

## Figures and Tables

**Table 1:** Descriptive analysis.

Variable	Mean	Range	Standard Deviation	Median	Mode	Variance
Tumor stage	3.5	3–4	0.5	4	4	0.25
Age	63.98	52–81	6.71	63	69	63.93
BMI	25.55	16.16–32.89	4.63	26.005	23.01	25.97

**Table 2:** Characteristics of the patients.

Characteristic	Total Sample	Intervention	Control	*p* Value
Age, mean, standard deviation (STD)	63.98 (STD 6.71)	64.04 (STD 6.78)	63.93 (STD 6.72)	0.94
Male sex	74	38	36	0.64
Tumor stage				0.64
3	44	21	23	
4	48	25	23	
BMI, mean, standard deviation	25.55 (STD 4.63)	24.94 (STD 4.1)	26.15 (STD 5.09)	0.25
FOIS baseline range	1 to 3	1 to 3	1 to 3	0.64
FOIS score 3 months (range)	1 to 5	2 to 5	1 to 3	<0.000
FOIS score 6 months (range)	1 to 6	4 to 6	1 to 5	<0.000
FOIS score 9 months (range)	2 to 7	5 to 7	2 to 6	<0.000
FOIS score 12 months (range)	3 to 7	6 to 7	3 to 7	<0.000
PSS H&N Eating in Public baseline (range)	0 to 25	0 to 25	0 to 25	1
PSS H&N Eating in Public 3 months (range)	25 to 75	50 to 75	25 to 50	<0.000
PSS H&N Eating in Public 6 months (range)	50 to 100	75 to 100	50 to 75	<0.000
PSS H&N Eating in Public 9 months (range)	50 to 100	75 to 100	50 to 100	<0.000
PSS H&N Eating in Public 12 months (range)	75 to 100	75 to 100	75 to 100	<0.000
PSS H&N Normalcy of Diet baseline (range)	0 to 20	0 to 20	0 to 20	0.33
PSS H&N Normalcy of Diet 3 months (range)	30 to 70	50 to 70	30 to 50	<0.000
PSS H&N Normalcy of Diet 6 months (range)	50 to 100	80 to 100	50 to 70	<0.000
PSS H&N Normalcy of Diet 9 months (range)	60 to 100	90 to 100	60 to 90	<0.000
PSS H&N Normalcy of Diet 12 months (range)	80 to 100	90 to 100	80 to 100	<0.000

## Data Availability

The data presented in this study are available in this article and on request from the corresponding author.
